# “I Have Grown Accustomed to Being Rejected”: EFL Academics’ Responses Toward Power Relations in Research Practice

**DOI:** 10.3389/fpsyg.2022.924333

**Published:** 2022-06-09

**Authors:** Hua Lu, Sook Jhee Yoon

**Affiliations:** ^1^School of Foreign Studies, Anhui Polytechnic University, Wuhu, China; ^2^Faculty of Education, Languages and Psychology, SEGi University, Kota Damansara, Malaysia

**Keywords:** power relations, EFL academics, research practice, research grant applying, academic writing and publishing

## Abstract

While there has been an increasing interest in English as a foreign language (EFL) teachers’ research engagement and researcher identity construction, scant attention has been paid to tensions caused by the issue of power relations in their research practice. This study draws on data from semi-structured interviews complemented with data from narrative frames and document analysis to examine the influence of power relations on the research practice of six EFL academics and their coping strategies at a Chinese university. The data analysis reveals that for the participants in the study, even though they were driven to be engaged in research practice by a combination of intrinsic and extrinsic motivations, they found that their research endeavors were undermined by the marginalized status of EFL researchers from non-elite universities, as imposed by the Chinese academic circle. Nevertheless, in the face of potential bias against their peripheral academic status, they exerted their agency with micropolitical literacy and tried to seek a way out of the unfavorable academic culture. As EFL teachers at regular universities are increasingly expected to be more research-active and research-productive, more attention and support are needed to facilitate their professional development and researcher identity construction.

## Introduction

In recent years, there has been a surge of interest in EFL teachers’ research practice (e.g., Allison and Carey, 2007; Bai and Hudson, 2011; Borg, 2009; Bai et al., 2013; Yuan, 2017; Peng and Gao, 2019; Yuan et al., 2020) and researcher identity construction (e.g., Xu, 2014; Long and Huang, 2017; Tran et al., 2017; Teng, 2019; Nakata et al., 2021; Bao and Feng, 2022). Previous works by Barkhuizen (2009), Trent (2012), and Taylor (2017) have shown that research engagement can promote language teachers’ teaching effects and contribute to their professional growth in research knowledge and skills, as well as contribute to their career advancement. Additionally, research has been given a top priority in many higher education institutions across the world that wish to improve their global ranking (Quimbo and Sulabo, 2014; Dai et al., 2021), particularly in the publish-or-perish academic culture (Lee, 2014; Bai, 2018). Therefore, university EFL teachers, like teachers in other disciplines, face mounting pressure to be research-active and research-productive (Borg and Liu, 2013; Yang et al., 2021a).

In many higher education institutions, English proficiency courses are offered as a breadth subject taken by students across the disciplines. The language instructors are employed based on their qualifications in teaching English and their high level of proficiency in English (Bai et al., 2014; Zhou and Zhang, 2016; Xu, 2020). Experience in research, albeit an added strength, is often not one of the main criteria for landing the language instructor position. Hence, there is an inherent mismatch between instructors’ experiences and the expectations to publish (Wang and Han, 2011; Huang and Guo, 2019) if the “publish or perish” concept is imposed upon these language instructors. This inherent mismatch has unfairly led language instructors to be at a disadvantage where research and publication-related achievements are concerned. These language instructors are labeled as an “academically marginalized community” (Liu and Borg, 2014, p. 288), loosely defined as those who have shown unsatisfactory research creativity and productivity (Dai, 2009). Such a marginal situation necessitates an examination of power relations in language instructors’ research practice, particularly those from non-elite universities with limited institutional resources. While previous studies have detected the constraints inflicted upon EFL teachers’ researcher identity construction by the unfavorable academic context (Barkhuizen, 2009; Liu and Borg, 2014; Long and Huang, 2017), few have conducted a further exploration of the specific influence of the issue of power relations on their research practice. In this study, we limit the scope to EFL academics from a common comprehensive university in China, where both established researchers and struggling research practitioners are present in the same department. This study aims to fill this gap by addressing the following two research questions:

1. What influence does the issue of power relations have on the research practice of university EFL academics?
2. How do university EFL academics deal with the issue of power relations in their research practice?

## Literature Review

### University English as a Foreign Language Teachers’ Researcher Identity

A great deal of previous research suggests that identity plays a central role in language teachers’ professional development because it helps teachers understand their work and make sense of their professional roles (e.g., Tsui, 2007; Liu et al., 2011; Trent, 2011, 2014; Farrell, 2012; Xu, 2012; Xu, 2016; Teng, 2020a; Yang et al., 2021b). As described as dynamic, fluid, and multifaceted (Beijaard et al., 2004), the construction of teacher identity involves a complex process through which individual teachers are engaged in various forms of practice, such as teaching and research, in their situated professional contexts (Yuan, 2017). It is not uncommon for teachers to constantly construct and reconstruct their identities to integrate their personal and professional dimensions in socio-institutional conditions (Beijaard and Meijer, 2017). One example is the work of Selvi et al. (2022), who employed collaborative autoethnography to unpack the complexities of identity among non-native English language teaching practitioners. Their work shows that initial beliefs and interactions with people and space can lead to constant changes in identities. Similarly, Molina (2022) reported that in the transnational English language teaching contexts, English language teachers’ work displayed complexities, and the fluid and multifaceted dimensions of their transnational identities could transcend generalizations and stereotypes as they interacted with situated circumstances. Awadelkarim (2022) also reported that academics often manifested their selfhood in their research writing. These works show that growth in self can lead to changes in identity as an English language teaching practitioner and how they perceive their identity as a researcher.

Consistent with teacher identity, EFL teachers’ researcher identity also displays features of being dynamic, complex, and multifaceted (Teng, 2018). It has been noted that EFL academics’ researcher identity construction is subject to not only personal factors such as research knowledge and skills (Dai, 2009; Xu, 2020), research self-efficacy (Boran, 2018; Nakata et al., 2021), research motivations (Yuan et al., 2016; Peng and Gao, 2019), but also a number of institutional (Yang et al., 2021a), and socio-cultural factors (Norton and Early, 2011; Xu, 2014). Extensive research has shown that many socio-institutional factors exert tremendous influence upon EFL teachers’ researcher identity construction, such as global and national academic culture (e.g., Zhang, 2014; Tian et al., 2016; Tran et al., 2017; Teng, 2019; Gao and Zheng, 2020), institutional research culture (e.g., Bai, 2018; Farsani and Babaii, 2019; Alhassan and Ali, 2020; Bao and Feng, 2022), and institutional research policies (e.g., Xu, 2014; Long and Huang, 2017; Yuan et al., 2020; Yang et al., 2021a; Yuan, 2021). These socio-institutional factors play either a conducive or a constraining role in academics’ research engagement. For example, Gao and Zheng (2020) found that the socio-political context, such as a country’s highly centralized system in education, may put academics in a research dilemma because it largely restrains academics’ autonomy to decide what to research and where to publish. Likewise, based on four university EFL teachers’ research experiences, Long and Huang (2017) reported that the institutional context was only conducive to teachers’ research engagement with reasonable and attainable research requirements, while it became unsupportive when its requirements seemed to be unattainable to academics.

Subject to various factors, the construction of researcher identity is a long and arduous process (Yuan, 2017), particularly for university EFL teachers in current academic contexts. Influenced by the trends of marketization (Mok, 2009), new managerialism (Deem et al., 2008), the performative culture (Perryman, 2009; Yang et al., 2021a), and the intention of enhancing their global competitiveness, many higher education institutions have adopted a research-oriented culture and put explicit requirements for research output in institutional policies such as recruitment, promotion, and key performance evaluation (Wang and Han, 2011; Bai et al., 2012; Long and Huang, 2017). University EFL teachers are thus socio-institutionally driven to be research productive in order to meet these requirements. This is typically reflected in the institutional key performance appraisal system carried out in many countries and regions, which evaluates academics’ research productivity mainly by the quality and quantity of scholarly publications and research grants at high levels (Sikes, 2006; Chetty and Lubben, 2010). As these research policies demand certain levels of research excellence to be fulfilled, EFL teachers often feel that they are too stressed and incompetent to meet these stringent requirements (Yuan et al., 2020). Moreover, their institutional context with unfavorable research culture has been found to be more of a constraining factor than a supportive one in their research work (Long and Huang, 2017). As a result, becoming a researcher has proven to be a difficult and demanding journey for EFL teachers, during which they frequently grapple with various tensions and challenges in their situated socio-institutional contexts (Barkhuizen, 2009; Liu and Borg, 2014). In a study done by Yang et al. (2021a), they reported that both negative and positive emotions contributed to the professional identity tensions, which led the EFL teachers to be the disheartened performer, the miserable follower, the strenuous accommodator, and the fulfilled integrator.

However, there is also research finding that EFL academics exercised their agency to be actively engaged in research activities despite their contextual constraints. For example, Yuan (2017) found that a novice language academic exercised his self-agency to survive the publishing game and develop his academic identity within socially defined contexts. Similarly, based on a case study on a language academic’s research experiences, Teng (2020a) reported that a sense of agency was necessary to help academics learn and participate in the academic community. Overall, EFL academics’ research practice is subject to various factors, including socio-institutional factors as well as individual factors. Under these circumstances, teacher agency plays a pivotal role in academics’ research ability building and professional development (Xu, 2020).

### Tensions and Power Relations in University English as a Foreign Language Teachers’ Research Practice

In addition to the institutional research requirements, for university EFL teachers, their marginalized academic status (Liu, 2009; Liu and Borg, 2014) and weak research tradition (Dai, 2009) also cause tensions and challenges in their research practice. To start with, there is a tension between their objective of doing research for pedagogical practice and their institution’s demand of doing research for publication. Studies found that a large number of EFL teachers were willing to be engaged in research that could bring benefits to their pedagogical practice while not caring much about whether their research could get published or not (Barkhuizen, 2009; Liu and Borg, 2014), given that most EFL teachers at tertiary institutions were initially recruited more as language instructors than competent researchers (Liu and Borg, 2014). However, in many institutions, the usual research requirements in institutional policies explicitly state the official stance of the institutional authorities: only published research counts as research (Wang and Han, 2011). This difference in the purpose of doing research highlights the conflicts between teachers’ intrinsic motivation and their extrinsic pressure to do research. While EFL teachers hold the view that the priority of research should be set on pedagogical benefits, their institutions set specific requirements for research output in quantified terms. This may eventually lead to a decline in academics’ intrinsic motivation to do research when they feel doing research is a compulsory duty externally enforced by their institution (Xu, 2014), which has absolute power over them.

As early as the 1980s, Foucault (1980) pointed out that power is pervasive in modern society and power relations are omnipresent. Power, according to Van Dijk (2013), is a property of relationships between social groups, institutions, or organizations. While not everyone has equal access to valued social resources, dominant groups or institutions may enact or legitimize power abuse and inequality in text and talk (Van Dijk, 2013). Society and culture are built on discourse (Fairclough et al., 2011). Being interpretative and explanatory, discourse analysis *per se* studies complex social phenomena with a multi-disciplinary and multi-methodological approach (Wodak and Meyer, 2009). It views language as social interaction and addresses social problems (Fairclough et al., 2011), one of which is professional and institutional power (Van Dijk, 2005). In specific social domains such as educational organizations, which possess a particular order of discourse (Fairclough, 2001), power and dominance are linked with the rules that serve as the background of the “discursive reproduction of power” (Van Dijk, 2005, p. 478) in such institutions. Members of other groups who are reliant on institutional power are the victims of such power. However, their dissenting discourses have received far less attention (Van Dijk, 2005). Foucault’s and others’ analyses of power and power relations have had a substantial impact on education (Dussel, 2010), since education is often associated with power (Gore, 1995). The issues caused by the omnipresence of power relations are particularly salient for university EFL teachers, who are victims of the tensions induced by power relations in their research practice. For example, both Braine (2005) and Yuan (2017) have noticed that EFL academics might experience bias against their research topics and research contexts. Confined to their local socio-cultural and educational contexts, EFL teachers’ research topics and focuses might not be taken as mainstream research interests, particularly given EFL teachers’ low academic status. Furthermore, some studies also mentioned that EFL academics might encounter bias in publishing articles when academic journals’ preferences are influenced by social networks (Xu, 2014; Yuan et al., 2020), the author’s academic status (Yuan et al., 2020), educational background (Dai et al., 2021), or professional title (Dai et al., 2021), which increases the tension in publishing their research work and thus induces some negative emotions such as complaint and disappointment (Xu, 2014; Yuan, 2021; Yuan et al., 2020). Given that the number of language academic journals is small compared to a large number of EFL teachers’ publishing needs (Xu, 2014), the dim reality aggravates the tensions related to power relations experienced by EFL teachers in their research engagement.

To date, while previous studies have brought the issue of power relations regarding EFL academics to people’s attention, the specific tensions caused by power relations in their research practice and how they cope with these tensions remain underexplored, particularly for those EFL teachers from non-elite universities without a prestigious research reputation. What potential bias and tensions they might encounter and how they deal with these tensions in research practice may be of broad relevance to language instructors in similar socio-cultural contexts across the globe. Therefore, adopting the theoretical lens provided by Foucault’s account of power relations (Foucault, 1980), this study aims to offer a nuanced understanding of this topic by exploring the influence of power relations on the research practice of EFL academics from a non-elite university and their coping strategies in the context of higher education in China.

## Methodology

### Research Context and Participants

The study was conducted at a common public university in central China. This university was chosen for two reasons. First, it is a typical non-elite public university with a middle ranking among all the higher education institutions in China. Public universities like this constitute approximately 94% of China’s higher education institutions (Wang, 2018). Two, given that the first author has a professional relationship with the university, it was chosen as the research site to ensure the completion of data collection based on factors of accessibility, feasibility, and familiarity (Hatch, 2002). In order to promote its ranking and further its development, this university has constantly adopted a research-oriented tendency in its institutional policies, even with the release of China’s national research policy, breaking the “five-only,” which intends to deemphasize the top priority of research for higher education institutions. Take the 3-year key performance appraisal system at this university, for example. It offers options of a teaching track, a teaching-research track, or a research track for teachers to take. However, the requirements for research output in the research track are so demanding and unattainable that no one actually chooses this option. As for the teaching track, only those with an “excellent” teaching evaluation title (top 20% in annual teaching evaluation get this title) in 3 consecutive years can pass with this option. In the most recent key performance appraisal from the cycle of 2019–2021, only 7 passed the teaching track among the 90 faculty members in the School of Foreign Studies. Therefore, the majority of teachers in this department still had to choose the teaching-research track, which imposes stringent and specific requirements on research productivity based on teachers’ professional titles, mainly manifested in research grants and article publications, in addition to certain teaching requirements. EFL teachers thus need to be actively engaged in research activities and have research outcomes as required regardless of their professional titles.

Adopting a qualitative research approach, this study used purposive sampling to select the participants (Merriam and Tisdell, 2015). For three reasons, six EFL teachers from the School of Foreign Studies at the research site were selected. First, regardless of whether they were established researchers or struggling research practitioners, they were all actively engaged in research practice and assumed the dual roles of EFL teacher and researcher at the same time. Second, they were all willing to provide rich information and share their stories due to their friendly relationship with the first author, which guaranteed the completion of data collection (Silverman, 2013). Third, they were at different professional phases and varied in different aspects, such as educational background, professional title, and research area, which helped achieve maximum variation among participants (Patton, 2014). The detailed background information of the participants is presented in Table 1. The six participants are referred to as T1, T2, T3, T4, T5, and T6.

**TABLE 1:** Background information of the participants.

Name	Gender	Age	Educational background	Professional title	Research area	Years of teaching
T1	Male	Late-30s	Ph.D. in foreign linguistics and applied linguistics MA in English linguistics and literature BA in English language education	Professor	Applied linguistics	18
T2	Female	Early-50s	Ph.D. in neurolinguistics MA in English linguistics and literature BA in English language	Professor	Neurolinguistics	25
T3	Female	Early-40s	MA in British literature BA in English language	Associate professor	British literature	13
T4	Female	Mid-40s	MA in English teaching pedagogy BA in English language	Associate professor	English teaching pedagogy	17
T5	Male	Mid-40s	Ph.D. in corpus linguistics MA in applied linguistics BA in English language	Lecturer	Corpus linguistics	14
T6	Male	Late-30s	MA in second language acquisition BA in English language	Lecturer	Second language acquisition	16

### Data Collection

This study drew data mainly from three sources, namely, narrative frame, semi-structured interview, and document analysis. At the beginning of the study, a narrative frame was used to collect basic and general information of the participant’s storied experiences (Barkhuizen, 2014). The narrative frame provided to the participants was adapted from the ones used by Xu (2014) and Teng (2019). The adapted narrative frame elicited not only the basic personal information from the participants, but also information related to their research practice, such as the participants’ research motivations, attitudes toward the institutional research requirements, perceived challenges in their research practice, and desired institutional research support.

Then, semi-structured interviews were conducted by the first author in a one-on-one and face-to-face manner with all the participants. To ensure the validity of the interview questions, prior to the interview data collection stage, we sent an email to invite one of the leading scholars on teacher education in Hong Kong as our expert reviewer to validate the interview protocol on our research. The expert generously granted our request and wrote detailed remarks on the original interview questions we sent him. He advised us to rephrase some questions so that they could probe into the teachers’ experiences. For example, “With regard to research, do you have any particular experiences to share with us?” or “What factors will influence your possibility of doing research? Any examples?” Based on the expert’s advice, we rephrased some of the interview questions as suggested. After that, the first author proceeded to the interview stage. In the interviews, the first author asked the participants some further questions concerning what they wrote in narrative frames to gain a clearer understanding of their responses. Then, the interview moved on to explore the participants’ research experiences, particularly the critical incidents or the most memorable events they have encountered, along with their emotions, reflections and comments. During the interviews, the participants were invited to air their opinions on the influencing factors in their research work and their corresponding feelings as well. For example, they were asked to reflect on their perceived challenges and tensions in their research work, analyze the possible reasons for these challenges, and develop potential solutions to them. The interviews lasted 40–60 min for each participant, and they were all conducted by the first author in Chinese (the mother tongue for both the interviewer and the participants). All the interviews were audio-recorded with the participants’ permission and transcribed verbatim. After that, the transcriptions were sent back to the participants for accuracy checking.

Also, the institutional documents concerning teachers’ research output requirements were collected by the first author to provide additional information about the participants’ research contexts and settings (Bowen, 2009). Furthermore, these documents, which listed specific requirements for faculty members’ research output as well as a system of rewards and penalties, can be used to supplement and triangulate the collected interview and narrative frame data in order to answer the research questions. With the permission of the dean of the School of Foreign Studies, the first author collected the relevant institutional research documents at the research site.

### Data Analysis

Thematic analysis and content analysis were used in this study. While the former helped the authors analyze data collected from narrative frames and interviews, the latter was used to analyze collected institutional research documents. A qualitative, inductive approach (Miles et al., 2018) was adopted in thematic data analysis. The process is as follows. First, we repeatedly read the interview transcripts and the participants’ written narrative frames to familiarize ourselves with the collected data. Second, during the process of data review, particular attention has been paid to the possible evidence of the issue of power relations (e.g., tensions, obstacles, and potential bias caused by power relations) in the participants’ research practice and the participants’ responses to this issue. This process of open coding resulted in a wide range of codes, such as “stringent institutional research requirements,” “limitations of the institutional platform,” “peripheral academic discipline status,” “reviewing experts’ disciplinary tendencies,” “journal editors’ preferences,” “emotional acceptance of the disadvantaged status,” and “exercising self-agency to enhance research competence.” All these codes were further compared and integrated to produce the themes that represented the venues (e.g., research grant applications and academic publishing) and sources of the power relations (e.g., the academic circle, reviewing experts, journal editors, and the institution) in the participants’ research work. As a result, two themes emerged from these codes, that is, “complexities in research grant applying” and “struggles in academic writing and publishing.” As for the participants’ coping strategies, “exercising teacher agency with micropolitical literacy” was the emerged theme since their emotional responses and behaviors of exercising self-agency toward the tensions caused by power relations were their agency-driven actions with micropolitical literacy. To enhance the trustworthiness of the study, the second author, a qualitative researcher with a Ph.D. in education, was invited to analyze the data as well. The first author and the second author then went through several discussions about the disagreements in the codes, eventually reaching an inter-rater agreement of more than 90%.

## Findings

A review of the institutional research policies indicates that the university mainly places requirements for research output on research grants and scholarly publications. The participants therefore put their efforts into these two directions to meet the institutional requirements. In both their narrative frames and interviews, they frequently talked about the challenges, obstacles, and tensions they have experienced in research grant applying and academic writing and publishing, along with their emotions and actions in dealing with the tensions in their research practice.

### Experiencing Complexities in Research Grant Applying

As one of the main criteria of teachers’ research productivity, research grants appear in every research policy at the participants’ workplace. Applying for research grants was one of the institutional expectations of the participants’ research practice (T2, T3, T4, narrative frames) and their constant research endeavors in these years (T1, T5, T6, narrative frames). For some of them, getting a research grant, particularly a high-level one, was a critical event in their research journey. T1, a prolific researcher with the professional title of professor, only considered himself “a real researcher” when he successfully got a research grant from the National Social Science Foundation, one of the highest level grants in China, in 2011 (T1, interview). Similarly, T2 started to build her research team in 2016 because she felt she had “a responsibility” after securing the national research grant that year (T2, interview). However, in their department, getting a national research grant was extremely rare. Back then, their successes were reported as breaking news on the university’s website.

Despite a few participants’ successful experiences, most participants expressed their frustrations and anxieties in applying for research grants, which were often derived from the potential bias in academia, in addition to their own limited research competence. While they admitted that they needed to improve “the unsatisfactory quality of the research grant applications” (T5, interview) they wrote, they found that they also had to face the bias against their academic discipline in some provincial research grants. T4, a conscientious EFL teacher with a research interest in English teaching pedagogy, shared her disheartening experience of grant application.

“When we are applying, we may be restricted to some research grants. For example, the Provincial Social Science Foundation only values theoretical research and allows those who do that to apply. We conduct research on teaching, teachers, or students, and our research is regarded as teaching research and is not allowed to apply” (T4, interview).

Because many EFL teachers’ primary concern was teaching, their research interest was naturally in teaching research, which was of practical-oriented value to them. However, the restriction on teaching research in grant applications meant that they had an even smaller chance of getting grants, which discouraged their research engagement and enthusiasm. They felt that they needed to be careful with their research focus to increase the chance of being successful.

The potential bias against teaching research and the importance of research topics were also echoed in T6’s story. When sharing his experience of a successful research grant application with the first author in the interview, he candidly admitted that it was a “coincidental and fortunate” event for him because the application he wrote was a study on students’ spoken English ability, which was not valued as a theoretical study in academia. Research grant applications on English teaching pedagogy like this were not favored at the provincial level. His grant application was immediately turned down when he applied for a provincial grant. Nevertheless, he continued to send the same application to another research grant, and it passed with the reviewing expert’s approval. He commented,

“This thing (getting research grants) is really hard to tell. It is hard to tell because when the same proposal is sent to different experts, some may find it meaningless, others may like it very much. There are the factors of luck and gambling involved” (T6, interview).

While the unpredictable factor in T6’s research grant application was experts’ preferences, for T3, it was a researcher’s academic status. As a diligent researcher, T3 has been actively applying for research grants at various levels since she returned to her work institution from a visiting scholar program in Shanghai in 2016. She believed she had reached the peak of her research after extensive reading and contact with the most cutting-edge knowledge in her field. Therefore, she applied for the National Social Science Foundation grant with passion and confidence. However, after a couple of tries, she found that an established scholar got the national research grant on a very similar research topic to hers when she was applying for the third time, which was devastating for her because she knew it meant “no hope” for a young researcher like her to get this grant if she continued to “follow the same research direction” (T3, interview). She lamentably concluded,

“In grant applying, maybe sometimes others have quicker research results than us. Like when I applied for the national research grant, that scholar got the grant a little bit earlier. It is definitely a hindrance for us liberal arts researchers” (T3, interview).

In addition to the unfavorable factors analyzed above, in 2021, the participants’ work institution issued a new research policy stating that only those who have experience in applying for the national research grant are allowed to apply for the provincial higher education research grant. This policy puts many EFL lecturers over 35 without a doctoral degree in a Catch-22 dilemma. On the one hand, they need a provincial research grant like this to get a senior professional title. On the other hand, only those who have a senior professional title, a doctoral degree, or are under 35 are eligible to apply for the national research grant according to the national policy. Given that more than half of the faculty members in the English department are lecturers in their late thirties or early forties without a doctoral degree, this institutional policy has worsened the situation of the whole department because it decreases many frontline teachers’ chances of getting provincial research grants. As one of the participants commented,

“The current policy is to see what level of research grants we get, but in fact, we all know some high-level research grants, ordinary teachers don’t have a chance to get them at all, because not everyone is allowed to apply for them in the first place. This is actually unfair” (T5, interview).

While getting research grants meant “recognition from academia” (T1, interview), the process of applying turned out to be entangled with complex power relations issues such as potential bias against the academic discipline, experts’ preferences, researchers’ academic status, and the institutional policy, which increased the tensions and difficulties in grant applications for the participants. Given the stringent institutional requirements on the level and number of research grants, the participants felt they were always under immense pressure and experienced intense anxiety when they were constantly striving for successful grant applications.

### Experiencing Struggles in Academic Writing and Publishing

According to the participants, they were doing research not only for their own “research interests and joy” (T1, T2, T3, T4, T5, narrative frames), but also for external motivations such as “promotion in professional titles” (T1, T2, narrative frames; T4, interview), and “passing the key performance appraisal” (T6, narrative frame). In some cases, the role played by external motivations was much greater than that of intrinsic motivations. T6, for example, repeatedly emphasized the external pressure of having research output. He claimed that he was writing manuscripts purely to meet the institutional requirements; otherwise, he would not do it because the process was “torturing and painful” (T6, narrative frame). Despite his reluctance and painful feelings, he persisted in writing manuscripts because it was “a system constraint” (T6, interview).

Even though mid-career lecturers like T6 felt it was enormously stressful to be engaged in research, the pressure of having research productivity was actually on all the participants, regardless of their professional titles. Tensions resulting from power relations were also evident in the research practice of academics with senior professional titles. In both his narrative frame and interview, T1, a rising academic who secured a full professorship in his thirties several years ago, mentioned the heavy pressure of publishing in top-tier journals more than once. According to the university’s research policy, professors like him needed to have scholarly publications in Chinese Social Science Citation Index (CSSCI) or Social Science Citation Index (SSCI) journals to pass the key performance appraisal. Thus, he felt frustrated and anxious when “it’s getting harder and harder to publish papers in high-level journals” (T1, narrative frame). Compared with his smooth publishing experiences in his Ph.D. studies at a prestigious university, he attributed part of his setbacks and frustrations in publishing to the platform of his current work institution.

“The school’s platform is limited because it is not among the universities of Project 211 and 985, which means it provides us with insufficient space for academic development. When I was a Ph.D. candidate at University X (pseudonym), I basically got papers published in all the CSSCI foreign language journals. But now, I think the quality of my manuscripts may be better than before, but the difficulty of being accepted by the CSSCI journals has increased. I think a large part of the reason is the limitations of the platform. The editors have a certain judgment when they see the name of our school” (T1, interview).

While the platform of the non-elite university proved to be a discouraging factor in academic publishing, the participants also noticed that the power of social networks had similar negative impact on them. As previous studies have pointed out, social networking sometimes played an important role in publishing in some Chinese foreign language journals, which tended to favor manuscripts from their acquaintances or established scholars rather than follow the blind review system (Xu, 2014; Yuan et al., 2020). This might increase difficulties in publishing for EFL academics without prestigious academic status or strong social networks. Based on years of submitting manuscripts to top-tier journals, T2, a newly promoted professor with publications in one CSSCI journal and several SSCI journals, made such comments,

“I feel that the academic circle culture of foreign language journals, especially high-level journals in China, is still too important. But there is no such thing in foreign journals of neurolinguistics. Comparatively speaking, they are fair and transparent” (T2, interview).

In addition to the academic circle culture, in some Chinese journals, editors have great power in selecting submitted articles. This could also pose challenges to some EFL academics whose research topics might not be appreciated by the editors. As T6 recalled, once he applied the teaching method of the flipped classroom in his course, then he wrote an article about the improved teaching effects based on the application, but his manuscript was immediately rejected after initial screening because “that teaching method is out of date” according to the editor of the journal he submitted (T6, interview). He felt that the ultimate reason for the rejection was the editor’s preference.

“The journal I submitted my manuscript to is a non-CSSCI journal. Still, the editors-in-chief of these journals are very picky now. Too many manuscripts are submitted to them every day. He glances at the research topic to see if it’s new or if he is interested in it. Their preferences matter so much” (T6, interview).

As a result, T6 felt there was practically little hope for him to have professional growth in terms of research achievements. Given that the platform provided by the university was so mediocre that there would be “no invitations from journals for manuscripts” (T6, interview), it was very challenging for EFL researchers like him without senior professional titles or a doctoral degree to get manuscripts published in CSSCI journals. Even some non-CSSCI journals reject his manuscripts due to a lack of interest in his research focus on English language teaching. T6 felt that he was “struggling at the bottom of academia” (T6, interview) as an EFL teacher at a non-elite university in academic publishing. In a similar vein, T5, a senior lecturer with a Ph.D. who had persevered in doing research for many years, expressed a similar sense of struggle when he made comments on the insufficient institutional support in his research practice.

“The big environment can’t help much. No supporting policies, no institutional supporting resources, nothing. I think the big environment, to be honest, the department didn’t help me at all. Basically, I am struggling desperately alone” (T5, interview).

Facing the dim reality of publishing in prestigious journals such as CSSCI journals, the participants felt that they were struggling helplessly with their peripheral academic status as EFL teachers from non-elite universities. They admitted that sometimes their failures in publishing were because “the quality of the manuscript was not good enough” (T4, interview) or “it was of low quality” (T5, interview). However, they also experienced the obstacles inflicted on them by the issue of power relations from the academic circle, the journal editors, or the institutional platform. Their disadvantaged status as marginalized academics from a non-elite university intensified the tensions in their publishing experiences. A combination of various factors led to their frequent failures in academic publishing. As T4 shared,

“At the beginning, when my applications were unsuccessful or my papers were rejected, I felt that the blow was quite large. But now, maybe I’ve had too many failures. I think I have grown accustomed to being rejected” (T4, interview).

### Exercising Teacher Agency With Micropolitical Literacy

The participants’ years of participation and reflection in research grant applications and academic publishing aided in the development of their micropolitical literacy (Kelchtermans, 2005), which guided their ongoing research practice as striving and struggling EFL academics. Based on the participants’ responses, on the one hand, they admitted that “it is human nature to feel depressed” (T1, interview) when they suffered from failures in grant applications and manuscript publications. On the other hand, they calmly accepted that there may be “some uncertain factors, such as reviewers’ preferences” (T3, interview) causing potential bias against their research work in the process. Focusing on her research area of British literature, T3 diligently applied for the national research grant and others in this field on a regular basis. In spite of her many failures in grant applications, she rationally commented,

“It is normal that people don’t see eye to eye regarding grant applications. Maybe the reviewers have disciplinary tendencies. In terms of practicality, applications relating to translation definitely hold a certain advantage over those of literature. There is also linguistics; they are more pragmatic in the first place. Then they may have a certain advantage on the reviewers’ side, and I can understand this” (T3, interview).

The above quote suggests the participants’ awareness of the existence of the issue of power relations and its impact on their research practice. As part of their micropolitical literacy, such an awareness helped them deal with the tensions caused by power relations in a rational manner. Instead of solely blaming the potential bias and feeling sorry for themselves, the participants focused on improving themselves to increase the chances of getting more research outcomes. This was most likely influenced by their pragmatic mindset for success in the performativity (Perryman, 2009) and publish-or-perish culture (Lee, 2014). As T6 stated,

“Only when your manuscript is published can you prove your success. If you write a lot, but you can’t get them published, then it’s all useless. Nobody cares about your research and gives you their approval” (T6, interview).

Evident in the above quote was the participants’ desire for research outcomes. Socio-culturally and institutionally driven, the participants naturally set research outcomes as the goal of their endeavors. Their micropolitical literacy made them aware that basically there was nothing they could do about their academic discipline’s marginal status; thus, they exerted their agency to take proactive steps to improve their research competence. For example, some participants admitted that they lacked systematic doctoral training or sufficient research knowledge and skills to write high-quality research grant applications and manuscripts. As T4 revealed,

“In terms of difficulty in research, I think my theoretical knowledge is very weak because I basically read theories by myself, and no one guides me on how to understand them. I think there must be some misconceptions in my understanding, and even some that are completely wrong. I think this is probably a very important reason why there has been no big breakthrough in my research for so many years” (T4, interview).

Therefore, hoping to improve the theoretical understanding of English teaching pedagogy, she applied for an overseas Ph.D. program in education. In this way, she felt that she could improve her research competence by receiving systematic academic training in the forthcoming Ph.D. studies. In addition, many participants frequently attended lectures on research grant applications and academic writing given by leading experts in academia to keep up with the frontline trends in EFL research. In narrative frames, more than half of the participants expressed their wish to “invite more experts to deliver seminars and lectures” in terms of institutional support to improve their research competence (T1, T3, T4, T6, narrative frames).

As for the participants with a doctoral degree, the common practice they adopted was to constantly revise their research grant applications and manuscripts on their own or seek constructive comments from other academics. When asked about how he handled the failures in grant applications and academic publishing, T1 emphasized that the most important thing was to locate the deficiencies and make improvements.

“Take time to locate the deficiencies in our research grant applications and manuscripts. Then carefully try to figure out which problems can be solved by ourselves and which can be solved by asking for help from others, so that we can solve the problems as much as possible to polish and improve the quality of our work” (T1, interview).

This self-improving practice was also shared by T5, another participant with a Ph.D. who felt that “writing a research grant application is like cooking” (T5, interview). He further elaborated,

“Writing a research grant application is not the same as writing a thesis. Writing an application is like writing a research plan, right? After you write the application, if you put it away for a week or two and read it again, you will feel that the original writing is not satisfactory and you can improve it. Because writing a research grant application is like cooking, it can never be done too carefully” (T5, interview).

Confronting the potential bias and tensions caused by power relations in the Chinese academic circle, the participants also tried to seek an alternative as a way out of the contextual constraints. For example, having experienced the different degrees of difficulties in publishing in local and international journals, T2 began to shift her focus to submitting manuscripts to SSCI journals. She recalled that, despite the fact that publishing in international journals was also difficult, the reviewers’ comments were very constructive, which helped “increase the rigor” of her manuscripts (T2, interview). In contrast to T2, T1 adopted a more balanced approach to academic publishing. While he kept submitting to CSSCI journals at the current rate of several times a year, he began to submit English manuscripts to international journals to increase his chance of having scholarly publications in prestigious journals. In 2021, he managed to get one manuscript published in a CSSCI journal and one in an SSCI journal as a result of collaborative writing with an academic from another university. No matter what form of agency the participants took, these were all their endeavors under the influence of micropolitical literacy to have as many research outcomes as they could to meet the institutional requirements.

## Discussion

Echoing Foucault’s account of power relations (Foucault, 1980), this study found that power relations were omnipresent in the research practice of EFL academics, an academically marginalized group, regardless of their professional titles. Professors, associate professors, and lecturers all reported experiencing tensions in power relations to varying degrees. Moreover, consistent with previous studies (Borg and Liu, 2013; Yuan, 2017; Yang et al., 2021a), this research found that the participants were under immense pressure to be engaged in research in their situated socio-institutional context. Under the influence of the publish-or-perish (Lee, 2014) and performativity culture (Perryman, 2009), their work institution imposed stringent research requirements not only in the promotion system, but also in the key performance appraisal system. As a result, like academics from other disciplines, the participants who were traditionally focusing on EFL teaching needed to be actively engaged in research and have required research outcomes for career advancement. However, as some previous research pointed out, EFL teachers’ marginalized academic status might bring about hindrances to their research engagement and productivity (Liu, 2009; Liu and Borg, 2014; Yuan, 2021). The participants in this study also experienced the potential bias and tensions induced by their disadvantaged status as a marginalized academic community in higher education.

To comply with stringent institutional requirements on research grants and scholarly publications, the participants in this study were mainly engaged in these two research activities. The potential bias and tensions caused by power relations they encountered were, accordingly, in these two aspects. When in publishing, some tensions experienced by the participants were derived from the potential bias against their institutional platform, the unfavorable treatment of the journal editors’ preferences on research topics, and their lack of strong social networks in academia. While some of these research findings have been reported in previous studies, such as the center academia’s dismissal attitude toward EFL academics’ research topics and contexts (Braine, 2005; Yuan, 2017), the important role played by social networking in publishing when there was a lack of a transparent and fair double-blind review system (Xu, 2014; Yuan et al., 2020), the potential bias against the platform of academics’ work institutions has been seldom mentioned in existing literature. This is probably because insufficient attention has been paid to the group of EFL academics from non-elite universities. While previous studies reported that general socio-institutional culture played a significant role on EFL teachers’ research practice (Xu, 2014; Negash et al., 2019; Yuan, 2021; Yuan et al., 2020; Yang et al., 2021a; Bao and Feng, 2022), this study found that the specific factor of power relations exerted an undeniable negative impact on the research productivity of EFL academics from non-elite universities. Given that this particular group constitutes the majority of EFL academics (Wang, 2018), this research finding contributes to our understanding of the obstacles and potential bias caused by power relations that common EFL academics face in their research practice.

This study also found that there were tensions caused by power relations in the participants’ research practice of research grant applications. In the process of applying for various grants, they have encountered tensions induced by the potential bias against teaching research focuses and topics, reviewing experts’ preferences, and applicants’ academic status. However, this research finding of potential bias in academics’ research grant applications seems to have not been previously reported. A possible explanation for this may be the participants’ situated socio-institutional contexts. While getting manuscripts published in prestigious journals seems to be a universal research requirement in almost all higher education institutions around the world (Lee, 2014; Tian et al., 2016; Yuan, 2021; Yuan et al., 2020), some universities additionally list requirements for research grants in their institutional policies. Subject to the trends of marketization (Mok, 2009) and new managerialism (Deem et al., 2008), higher education institutions in China value the facilitative role played by research grants, especially the high-level ones, in promoting institutional development. The participants’ work institution is no exception. In fact, given that it is a non-elite public university with insufficient funding and resources, getting as much funding as possible through research grants seems to be a practical exercise to adopt. Situated in the culture of performativity and accountability (Perryman, 2009), the participants naturally follow the institutional requirements to focus on research grant applications in spite of their slim chances of being successful.

Even though the participants in this research experienced the potential bias caused by power relations in their research practice, surprisingly, most of them were found to accept this phenomenon with calmness, which is contrary to what has been found in previous studies. Prior research reported that potential bias in academia could trigger academics’ negative emotions such as complaint (Xu, 2014), upset (Yuan, 2021), disappointment (Yuan et al., 2020), and self-doubt (Yuan et al., 2020). However, the findings of the current study do not support the previous research. The participants did not indulge in negative emotions; instead, they cultivated their micropolitical literacy (Kelchtermans, 2005) and exercised teacher agency as a coping strategy to find a way out of their current unfavorable situation. This rather interesting finding might be related to the participants’ self-positioning and pragmatic mindset. Being EFL teachers at a non-elite university, they were aware of their institution’s middle ranking and their marginalized academic status. One distinct example was T6, who used a metaphor to describe their status; they were struggling “at the bottom of the pyramid in academia” (T6, interview). The feeling of struggling and failing in research practice was so common that they had become “accustomed to being rejected” (T4, interview). Having said that, driven by a pragmatic mindset for research outcomes as career success, the participants continuously exerted their agency to improve their own research competence with the intention of achieving as many research outcomes as required. The challenges and setbacks in their unfavorable research environment actually activated their psychological and cognitive resources to a certain degree (Xue, 2021).

Therefore, echoing previous research results (Yuan, 2017; Teng, 2020b; Xu, 2020), teacher agency was found to be a crucial factor in the participants’ coping strategies to be more research engaged and research competent. However, the participants’ specific coping strategies concerning the potential bias of power relations varied. While some focused on enhancing their own research competence by attending academic lectures, enrolling in a doctoral program, and revising their manuscripts and research grant applications, others began to submit manuscripts to international journals as an alternative way out of the contextual constraints. These individualized coping strategies were also part of their micropolitical literacy, which not only helped them look at their marginalized academic status in a rational manner, but also helped them survive the potential bias of power relations in their research practice.

## Conclusion and Implications

This study explores the influence of power relations on the research practice of EFL academics from a non-elite Chinese university. It found that while EFL academics were driven to be research-active and research-productive by intrinsic and extrinsic motivations, they encountered tensions and potential bias in power relations against their marginalized academic status in academia. Despite that, EFL academics exercised self-agency with micropolitical literacy to find a way out of the unfavorable environment. The contribution of the study lies in exploring perspectives on the issue of power relations in the research practice of EFL academics from a non-elite university without research prestige or sufficient support, which represents the general situation of the majority of EFL academics in China. Therefore, the research findings may be of relevance to academics in similar contexts around the world. Further, it sheds light on common EFL academics’ research experiences in relation to their self-agency and external factors such as institutional requirements and the issue of power relations, which may also exert significant influence on EFL academics’ research practice and research productivity.

This study has some practical implications for EFL academics from non-elite universities in unfavorable socio-institutional contexts. First, in the publish-or-perish and performativity cultures, it is important for EFL academics to cultivate micropolitical literacy to be emotionally prepared for the possible setbacks and bias of power relations in the challenging process of research practice. Such micropolitical literacy could help them be aware of the potential bias at the socio-institutional level with a rational mentality. Given that their marginalized academic status cannot be changed overnight, there is also a need for EFL academics to exercise self-agency to enhance their research competence as a practical way out of the contextual constraints.

To promote EFL teachers’ research competence, universities may consider providing material support and inviting leading experts in academia to deliver frequent training and lectures on how to conduct research. By providing such systematic guidance and research assistance, universities can not only facilitate EFL teachers’ research competence through training and communication with experts, but also help them foster a sense of belonging in a supportive community. For non-elite universities with limited institutional resources, they may also consider cultivating a partnership with those research-intensive universities. In this way, the disadvantaged group of EFL academics may have a better chance of academic success if they can collaborate with their counterparts at prestigious universities.

Furthermore, given the marginalized academic status of EFL teachers and the potential bias of power relations in their research practice, university administrations and institutional policymakers may need to adopt encouraging and flexible policies to recognize EFL academics’ research efforts rather than simply impose stringent requirements on their research output. For example, non-elite universities could take measures to give EFL researchers credit for their efforts in applying for research grants instead of only acknowledging the secured ones. In this way, EFL academics may stay perseverant and motivated in research engagement in the current culture of performativity in higher education.

This study also has two limitations. First, the study only focused on six EFL academics from a non-elite university with a middle ranking in China. Future research may select participants from various types of universities to make a comparative study on the issue of power relations in EFL academics’ research practice. Second, the participants in this study were already active academics since they have been engaged in research non-stop for years. Future research may take novice researchers as participants and explore how they deal with the issue of power relations in their specific socio-institutional settings.

## Data Availability Statement

The raw data supporting the conclusions of this article will be made available by the authors upon request.

## Ethics Statement

The studies involving human participants were reviewed and approved by School of Foreign Studies, Anhui Polytechnic University. The patients/participants provided their written informed consent to participate in this study.

## Author Contributions

HL was mainly in charge of research methodology, data collection, data analysis, and drafting and revision of the manuscript. SY contributed to data analysis and revision of the manuscript. Both authors contributed to the article and approved the submitted version.

## Conflict of Interest

The authors declare that the research was conducted in the absence of any commercial or financial relationships that could be construed as a potential conflict of interest.

## Publisher’s Note

All claims expressed in this article are solely those of the authors and do not necessarily represent those of their affiliated organizations, or those of the publisher, the editors and the reviewers. Any product that may be evaluated in this article, or claim that may be made by its manufacturer, is not guaranteed or endorsed by the publisher.
